# (*E*)-Ethyl *N*′-[1-(4-methoxy­phen­yl)ethyl­idene]hydrazinecarboxyl­ate

**DOI:** 10.1107/S1600536808031462

**Published:** 2008-10-04

**Authors:** Lu-Ping Lv, Jian-Wu Xie, Wen-Bo Yu, Yu-Hui Mao, Xian-Chao Hu

**Affiliations:** a Department of Chemical Engineering, Hangzhou Vocational and Technical College, Hangzhou 310018, People’s Republic of China; b Research Center of Analysis and Measurement, Zhejiang University of Technology, Hangzhou 310014, People’s Republic of China

## Abstract

The mol­ecule of the title compound, C_12_H_16_N_2_O_3_, adopts a *trans* configuration with respect to the C=N bond. The dihedral angle between the benzene ring and the hydrazinecarboxyl­ate plane is 13.82 (6)°. In the crystal structure, mol­ecules are linked into centrosymmetric dimers by N—H⋯O and C—H⋯O hydrogen bonds, and the dimers are linked together by C—H⋯π inter­actions.

## Related literature

For general background, see: Parashar *et al.* (1988 ▶); Hadjoudis *et al.* (1987 ▶); Borg *et al.* (1999 ▶). For a related structure, see: Lv *et al.* (2008 ▶).
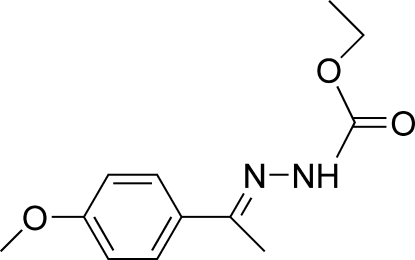


## Experimental

### 

#### Crystal data

C_12_H_16_N_2_O_3_
*M* *_r_* = 236.27
Orthorhombic, 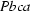

*a* = 12.1020 (11) Å
*b* = 8.1727 (7) Å
*c* = 25.476 (2) Å
*V* = 2519.8 (4) Å^3^
*Z* = 8
Mo *K*α radiation
μ = 0.09 mm^−1^
*T* = 123 (2) K
0.27 × 0.23 × 0.22 mm

#### Data collection

Bruker SMART CCD area-detector diffractometer
Absorption correction: multi-scan (*SADABS*, Bruker, 2002 ▶) *T*_min_ = 0.973, *T*_max_ = 0.981
12848 measured reflections
2222 independent reflections
1845 reflections with *I* > 2σ(*I*)
*R*_int_ = 0.026

#### Refinement

*R*[*F* ^2^ > 2σ(*F* ^2^)] = 0.040
*wR*(*F* ^2^) = 0.127
*S* = 1.07
2222 reflections
158 parameters
H-atom parameters constrained
Δρ_max_ = 0.19 e Å^−3^
Δρ_min_ = −0.14 e Å^−3^

### 

Data collection: *SMART* (Bruker, 2002 ▶); cell refinement: *SAINT* (Bruker, 2002 ▶); data reduction: *SAINT*; program(s) used to solve structure: *SHELXS97* (Sheldrick, 2008 ▶); program(s) used to refine structure: *SHELXL97* (Sheldrick, 2008 ▶); molecular graphics: *SHELXTL* (Sheldrick, 2008 ▶); software used to prepare material for publication: *SHELXTL*.

## Supplementary Material

Crystal structure: contains datablocks I, global. DOI: 10.1107/S1600536808031462/ci2686sup1.cif

Structure factors: contains datablocks I. DOI: 10.1107/S1600536808031462/ci2686Isup2.hkl

Additional supplementary materials:  crystallographic information; 3D view; checkCIF report

## Figures and Tables

**Table 1:** Hydrogen-bond geometry (Å, °) *Cg*1 is the centroid of the C2–C7 ring.

*D*—H⋯*A*	*D*—H	H⋯*A*	*D*⋯*A*	*D*—H⋯*A*
N2—H2*A*⋯O2^i^	0.86	2.10	2.914 (2)	157
C12—H12*C*⋯O2^i^	0.96	2.52	3.250 (2)	133
C1—H1*C*⋯*Cg*1^ii^	0.96	2.76	3.637 (2)	153

Symmetry codes: (i) 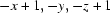; (ii) 
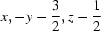
.

**Fig. 1.**
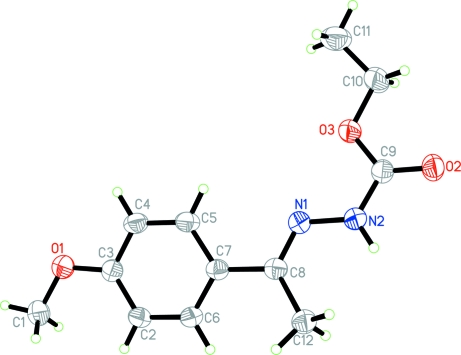
The molecular structure of the title compound, with the atom-numbering scheme. Displacement ellipsoids are drawn at the 30% probability level.

**Fig. 2.**
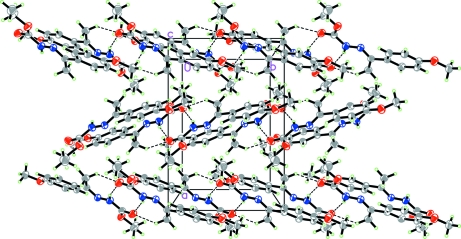
The crystal packing of the title compound, viewed approximately down the c axis. Hydrogen bonds are shown as dashed lines.

## supplementary crystallographic information

### Comment

Benzaldehydehydrazone derivatives have received considerable attention for a long time, due to their pharmacological activities (Parashar *et al.*, 1988) and their photochromic properties (Hadjoudis *et al.*, 1987). They are important intermidiates for 1,3,4-oxadiazoles, which have been reported to be versatile compounds with many properties (Borg *et al.*, 1999). As a further investigation of this type of derivatives, we report herein the crystal structure of the title compound.

The title molecule (Fig. 1) adopts a trans configuration with respect to the C═N double bond. The bond lengths and angles are comparable to those observed for (E)-methyl N'-[1-(4-methoxyphenyl)ethylidene]hydrazinecarboxylate (Lv *et al.*, 2008). Atoms C11 and C12 deviate from the O2/O3/N1/N2/C7-C10 plane by 0.406 (2) and 0.175 (2) Å, respectively. The dihedral angle between benzene (C2-C7) and O2/O3/N1/N2/C7-C10 planes is 13.82 (6)°.

In the crystal structure, intermolecular N—H···O and C–H···O hydrogen bonds (Table 1) link the molecules into centrosymmetric dimers (Fig. 2). A C—H···π contact (Table 1) between benzene ring (centroid Cg1) and C1-methyl group further stabilizes the structure.

### Experimental

4-Methoxy-acetophenone (1.50 g, 0.01 mol) and ethyl hydrazinecarboxylate (1.04 g, 0.01 mol) were dissolved in stirred methanol (25 ml) and left for 3.5 h at room temperature. The resulting solid was filtered off and recrystallized from ethanol to give the title compound (yield 83%, m.p. 465-468 K). Single crystals of the title compound suitable for X-ray analysis were obtained by slow evaporation of an ethanol solution.

### Refinement

H atoms were positioned geometrically, with N-H = 0.86 Å and C-H = 0.93, 0.97 and 0.96 Å for aromatic, methylene and methyl H, respectively, and constrained to ride on their parent atoms with U_iso_(H) = xU_eq_(C,N), where x = 1.5 for methyl H and x = 1.2 for all other H atoms. A rotating group model was used for the methyl groups.

### Crystal data

**Table:**

C_12_H_16_N_2_O_3_	*F*(000) = 1008
*M**_r_* = 236.27	*D*_x_ = 1.246 Mg m^−^^3^
Orthorhombic, *P**b**c**a*	Mo *K*α radiation, λ = 0.71073 Å
Hall symbol: -P 2ac 2ab	Cell parameters from 2222 reflections
*a* = 12.1020 (11) Å	θ = 1.6–25.0°
*b* = 8.1727 (7) Å	µ = 0.09 mm^−^^1^
*c* = 25.476 (2) Å	*T* = 123 K
*V* = 2519.8 (4) Å^3^	Block, colourless
*Z* = 8	0.27 × 0.23 × 0.22 mm

### Data collection

**Table:**

Bruker SMART CCD area-detector diffractometer	2222 independent reflections
Radiation source: fine-focus sealed tube	1845 reflections with *I* > 2σ(*I*)
graphite	*R*_int_ = 0.026
φ and ω scans	θ_max_ = 25.0°, θ_min_ = 1.6°
Absorption correction: multi-scan (SADABS, Bruker, 2002)	*h* = −12→14
*T*_min_ = 0.973, *T*_max_ = 0.981	*k* = −9→9
12848 measured reflections	*l* = −30→30

### Refinement

**Table:**

Refinement on *F*^2^	Secondary atom site location: difference Fourier map
Least-squares matrix: full	Hydrogen site location: inferred from neighbouring sites
*R*[*F*^2^ > 2σ(*F*^2^)] = 0.040	H-atom parameters constrained
*wR*(*F*^2^) = 0.127	*w* = 1/[σ^2^(*F*_o_^2^) + (0.0637*P*)^2^ + 0.6384*P*] where *P* = (*F*_o_^2^ + 2*F*_c_^2^)/3
*S* = 1.07	(Δ/σ)_max_ = 0.002
2222 reflections	Δρ_max_ = 0.19 e Å^−^^3^
158 parameters	Δρ_min_ = −0.14 e Å^−^^3^
0 restraints	Extinction correction: SHELXL97 (Sheldrick, 2008), Fc^*^=kFc[1+0.001xFc^2^λ^3^/sin(2θ)]^-1/4^
Primary atom site location: structure-invariant direct methods	Extinction coefficient: 0.0115 (13)

### Special details

**Table:**

Geometry. All esds (except the esd in the dihedral angle between two l.s. planes) are estimated using the full covariance matrix. The cell esds are taken into account individually in the estimation of esds in distances, angles and torsion angles; correlations between esds in cell parameters are only used when they are defined by crystal symmetry. An approximate (isotropic) treatment of cell esds is used for estimating esds involving l.s. planes.
Refinement. Refinement of F^2^ against ALL reflections. The weighted R-factor wR and goodness of fit S are based on F^2^, conventional R-factors R are based on F, with F set to zero for negative F^2^. The threshold expression of F^2^ > 2sigma(F^2^) is used only for calculating R-factors(gt) etc. and is not relevant to the choice of reflections for refinement. R-factors based on F^2^ are statistically about twice as large as those based on F, and R- factors based on ALL data will be even larger.

### Fractional atomic coordinates and isotropic or equivalent isotropic displacement parameters (Å^2^)

**Table:**

	*x*	*y*	*z*	*U*_iso_*/*U*_eq_	
C7	0.42346 (13)	0.5640 (2)	0.60487 (6)	0.0490 (4)	
C8	0.43416 (13)	0.4210 (2)	0.56947 (6)	0.0501 (4)	
C3	0.40760 (13)	0.8369 (2)	0.67166 (6)	0.0525 (4)	
C2	0.37209 (14)	0.8477 (2)	0.62024 (7)	0.0570 (4)	
H2	0.3430	0.9453	0.6075	0.068*	
C9	0.59258 (14)	0.0586 (2)	0.56465 (7)	0.0542 (4)	
C5	0.45905 (15)	0.5568 (2)	0.65717 (7)	0.0580 (5)	
H5	0.4890	0.4600	0.6700	0.070*	
C6	0.38006 (15)	0.7122 (2)	0.58781 (6)	0.0561 (4)	
H6	0.3555	0.7207	0.5533	0.067*	
C4	0.45058 (16)	0.6900 (2)	0.68974 (7)	0.0613 (5)	
H4	0.4740	0.6816	0.7244	0.074*	
C1	0.35816 (19)	1.1125 (2)	0.69025 (8)	0.0733 (6)	
H1A	0.4039	1.1577	0.6631	0.110*	
H1B	0.3550	1.1872	0.7193	0.110*	
H1C	0.2850	1.0948	0.6769	0.110*	
C11	0.7327 (2)	−0.0352 (3)	0.68377 (9)	0.0932 (7)	
H11A	0.7656	0.0694	0.6908	0.140*	
H11B	0.7815	−0.1204	0.6954	0.140*	
H11C	0.6637	−0.0435	0.7021	0.140*	
C10	0.7136 (2)	−0.0522 (3)	0.62760 (9)	0.0894 (8)	
H10A	0.6803	−0.1576	0.6201	0.107*	
H10B	0.7831	−0.0451	0.6087	0.107*	
O3	0.63987 (11)	0.07917 (16)	0.61102 (5)	0.0677 (4)	
O2	0.61004 (11)	−0.05717 (16)	0.53593 (5)	0.0701 (4)	
O1	0.40315 (12)	0.96202 (16)	0.70721 (5)	0.0673 (4)	
N2	0.52061 (12)	0.17888 (17)	0.55187 (5)	0.0571 (4)	
H2A	0.4835	0.1713	0.5232	0.068*	
N1	0.50534 (12)	0.31305 (17)	0.58366 (5)	0.0532 (4)	
C12	0.36482 (15)	0.4117 (2)	0.52056 (7)	0.0621 (5)	
H12A	0.4098	0.4361	0.4905	0.093*	
H12B	0.3056	0.4896	0.5228	0.093*	
H12C	0.3347	0.3036	0.5171	0.093*	

### Atomic displacement parameters (Å^2^)

**Table:**

	*U*^11^	*U*^22^	*U*^33^	*U*^12^	*U*^13^	*U*^23^
C7	0.0429 (8)	0.0533 (9)	0.0509 (9)	−0.0002 (7)	0.0003 (7)	0.0052 (7)
C8	0.0451 (9)	0.0546 (10)	0.0505 (9)	−0.0011 (7)	0.0025 (7)	0.0067 (7)
C3	0.0468 (9)	0.0571 (10)	0.0536 (9)	−0.0019 (8)	0.0000 (7)	−0.0028 (7)
C2	0.0595 (10)	0.0542 (10)	0.0572 (9)	0.0087 (8)	−0.0028 (8)	0.0067 (8)
C9	0.0501 (9)	0.0571 (10)	0.0555 (9)	0.0012 (8)	−0.0014 (7)	−0.0050 (8)
C5	0.0597 (10)	0.0560 (10)	0.0584 (10)	0.0046 (8)	−0.0103 (8)	0.0079 (8)
C6	0.0606 (10)	0.0615 (11)	0.0461 (8)	0.0065 (8)	−0.0038 (7)	0.0049 (8)
C4	0.0642 (11)	0.0664 (11)	0.0532 (9)	0.0029 (9)	−0.0134 (8)	0.0029 (8)
C1	0.0867 (14)	0.0597 (12)	0.0736 (12)	0.0091 (10)	−0.0031 (10)	−0.0111 (10)
C11	0.1006 (18)	0.0890 (16)	0.0900 (15)	0.0101 (14)	−0.0275 (13)	0.0137 (13)
C10	0.0913 (17)	0.0877 (16)	0.0894 (15)	0.0396 (13)	−0.0261 (12)	−0.0145 (12)
O3	0.0734 (9)	0.0651 (8)	0.0646 (8)	0.0184 (6)	−0.0172 (6)	−0.0106 (6)
O2	0.0664 (8)	0.0722 (9)	0.0715 (8)	0.0159 (7)	−0.0101 (6)	−0.0200 (7)
O1	0.0760 (9)	0.0648 (8)	0.0611 (7)	0.0067 (6)	−0.0071 (6)	−0.0088 (6)
N2	0.0598 (8)	0.0592 (9)	0.0522 (8)	0.0075 (7)	−0.0079 (6)	−0.0035 (6)
N1	0.0563 (8)	0.0512 (8)	0.0520 (8)	0.0021 (6)	−0.0004 (6)	−0.0005 (6)
C12	0.0575 (10)	0.0676 (12)	0.0613 (10)	0.0027 (9)	−0.0066 (8)	−0.0046 (9)

### Geometric parameters (Å, °)

**Table:**

C7—C6	1.390 (2)	C1—O1	1.412 (2)
C7—C5	1.401 (2)	C1—H1A	0.96
C7—C8	1.482 (2)	C1—H1B	0.96
C8—N1	1.285 (2)	C1—H1C	0.96
C8—C12	1.504 (2)	C11—C10	1.456 (3)
C3—O1	1.367 (2)	C11—H11A	0.96
C3—C2	1.382 (2)	C11—H11B	0.96
C3—C4	1.387 (2)	C11—H11C	0.96
C2—C6	1.385 (2)	C10—O3	1.458 (2)
C2—H2	0.93	C10—H10A	0.97
C9—O2	1.215 (2)	C10—H10B	0.97
C9—O3	1.323 (2)	N2—N1	1.376 (2)
C9—N2	1.353 (2)	N2—H2A	0.86
C5—C4	1.372 (2)	C12—H12A	0.96
C5—H5	0.93	C12—H12B	0.96
C6—H6	0.93	C12—H12C	0.96
C4—H4	0.93		
			
C6—C7—C5	116.71 (15)	O1—C1—H1C	109.5
C6—C7—C8	122.01 (14)	H1A—C1—H1C	109.5
C5—C7—C8	121.27 (15)	H1B—C1—H1C	109.5
N1—C8—C7	115.38 (14)	C10—C11—H11A	109.5
N1—C8—C12	124.94 (15)	C10—C11—H11B	109.5
C7—C8—C12	119.67 (14)	H11A—C11—H11B	109.5
O1—C3—C2	124.64 (16)	C10—C11—H11C	109.5
O1—C3—C4	116.25 (15)	H11A—C11—H11C	109.5
C2—C3—C4	119.11 (16)	H11B—C11—H11C	109.5
C3—C2—C6	119.53 (16)	C11—C10—O3	108.19 (18)
C3—C2—H2	120.2	C11—C10—H10A	110.1
C6—C2—H2	120.2	O3—C10—H10A	110.1
O2—C9—O3	124.14 (16)	C11—C10—H10B	110.1
O2—C9—N2	122.21 (16)	O3—C10—H10B	110.1
O3—C9—N2	113.64 (14)	H10A—C10—H10B	108.4
C4—C5—C7	121.26 (16)	C9—O3—C10	115.45 (14)
C4—C5—H5	119.4	C3—O1—C1	117.62 (14)
C7—C5—H5	119.4	C9—N2—N1	121.58 (14)
C2—C6—C7	122.46 (15)	C9—N2—H2A	119.2
C2—C6—H6	118.8	N1—N2—H2A	119.2
C7—C6—H6	118.8	C8—N1—N2	118.13 (14)
C5—C4—C3	120.91 (16)	C8—C12—H12A	109.5
C5—C4—H4	119.5	C8—C12—H12B	109.5
C3—C4—H4	119.5	H12A—C12—H12B	109.5
O1—C1—H1A	109.5	C8—C12—H12C	109.5
O1—C1—H1B	109.5	H12A—C12—H12C	109.5
H1A—C1—H1B	109.5	H12B—C12—H12C	109.5

### Hydrogen-bond geometry (Å, °)

**Table:**

*D*—H···*A*	*D*—H	H···*A*	*D*···*A*	*D*—H···*A*
N2—H2A···O2^i^	0.86	2.10	2.914 (2)	157
C12—H12C···O2^i^	0.96	2.52	3.250 (2)	133
C1—H1C···Cg1^ii^	0.96	2.76	3.637 (2)	153

Symmetry codes: (i) −*x*+1, −*y*, −*z*+1; (ii) *x*, −*y*−3/2, *z*−1/2.
